# Quality of Life Outcomes Following Organ-Sparing SBRT in Previously Irradiated Recurrent Head and Neck Cancer

**DOI:** 10.3389/fonc.2019.00836

**Published:** 2019-09-10

**Authors:** Emile Gogineni, Isabella Zhang, Zaker Rana, Mihaela Marrero, Gurtej Gill, Anurag Sharma, Adam C. Riegel, Sewit Teckie, Maged Ghaly

**Affiliations:** Department of Radiation Medicine, Northwell Health, New York, NY, United States

**Keywords:** SBRT, head and neck cancer, quality of life, stereotactic, radiosurgery, SABR

## Abstract

**Purpose:** To present a retrospective analysis of the efficacy, toxicity, and quality of life (QoL) of patients treated with OAR_*Extreme*_-sparing stereotactic body radiotherapy (SBRT) in previously-irradiated head and neck cancer.

**Materials/Methods:** From 11/2012 to 7/2015, 60 patients with in-field recurrence of head and neck cancer underwent re-irradiation with SBRT. Retreatment sites included the aerodigestive tract (43%), lateral neck (22%), and skull base (35%). The median prior RT dose was 63.6 Gy with a median time from prior irradiation of 16.5 months. The median volume treated was 61.0 cc. Patients were treated with 40 Gy in the definitive setting or 35 Gy in the post-operative setting in five fractions. Dose constraints to the OAR_*Extreme*_ were calculated with a BED calculator using an alpha/beta ratio of 3 to reduce the risk of late toxicities. QoL data was collected from patients at the time of consultation and at subsequent follow up appointments using the MD Anderson Dysphagia Inventory (MDADI) and Symptom Inventory (MDASI).

**Results:** The 1- and 2- year rates of local, regional, and distant control and overall survival were 79/79, 74/70, 74/71, and 59/45%, respectively. Late grade 3 toxicities were seen in 3% in the group treated to the aerodigestive tract and 1% in the group treated to the skull base. No grade 4 or 5 toxicities were observed. Patients with skull base re-irradiation maintained a stable QoL score after radiation treatment, while patients treated to the aerodigestive tract demonstrated a slight impairment associated with worsening dysphagia, compared to their pretreatment baseline. All groups experienced an increase in xerostomia.

**Conclusions:** OAR_*Extreme*_-sparing SBRT is able to achieve excellent tumor coverage while protecting the organs at highest risk of re-irradiation-related complications. The potential for lower toxicities and maintained QoL with this treatment makes it a promising option for salvage of recurrent head and neck cancer.

**Summary:**

Local control and overall survival rates for recurrent head and neck cancer remain poor, despite the use of local therapy. In addition, re-irradiation with conventional radiation therapy confers a high rate of grade 3 and higher late toxicities. SBRT appears to improve the therapeutic ratio in this patient population, and treatment planning with a focus on sparing OAR_*Extreme*_ may further decrease the rates of morbidity in these patients.

## Introduction

Locoregional recurrence within a previously-irradiated field represents a significant barrier in the long-term control of head and neck cancer, with a high rate of morbidity and mortality associated with local disease. Maximal surgical resection at the time of recurrence continues to represent the standard of care with adjuvant radiation considered when the risk of further recurrence is extremely high. Regardless, most patients are not surgical candidates due to their extent of tumor recurrence or other comorbidities limiting their ability to tolerate the operation (1). Both chemotherapy and conventionally fractionated radiation therapy have been used for recurrent, unresectable disease, with a median survival ranging from 6 to 9 months, with rates of grade 3 and higher late toxicities approaching 40% (2, 3).

Over the last decade, stereotactic body radiation therapy (SBRT) has been increasingly used for irradiation of recurrent head and neck cancer, both in the definitive and adjuvant settings. Recent studies have demonstrated the relatively low rates of severe toxicities with SBRT in the re-irradiation setting, but without a substantial improvement in overall survival compared to conventionally fractionated radiation therapy (4–6).

The purpose of aggressive locoregional recurrence is two-fold: first, locoregional disease is a leading cause of mortality in head and neck cancer and treatment of the recurrence may prolong survival (7). Second, tumor recurrence can cause significant symptoms, including pain, dysphagia, and cranial nerve deficits, which can be reduced by radiation. The goals of local control and palliation must then be balanced against the potential long-term risks of re-irradiation that could impair quality of life (QoL). In this study, we present the results of re-irradiation of in-field recurrence of head and neck cancer using SBRT while prioritizing specific normal tissue tolerances. The protection of normal tissues has become increasingly important as technology and patient outcomes improve and patients present with locoregional disease months or years after re-irradiation, and may require a second course of retreatment.

## Materials and Methods

**Figure 1:**
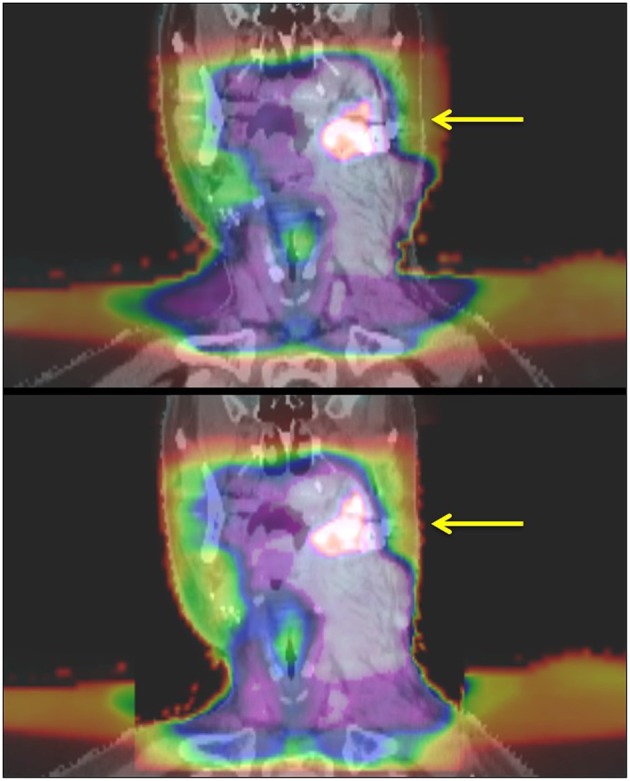
Deformable registration of initially delivered radiation doses onto retreatment planning CT. The yellow arrow highlights the PET-positive tumor. The top panel shows fusion of doses onto CT based on anatomical landmarks before deformable registration. The bottom panel shows deformably registered images with better delineation of doses delivered to normal tissue structures.

Approval was obtained from the CSSRC (Cancer Services Scientific Review Committee) and the IRB (IRB #15-089). The Informed Consent and HIPAA authorization were waived, given that it was a retrospective chart review and the fact that no PHI was reported. Sixty patients were treated in our institution with re-irradiation to a previously irradiated site—either in the definitive or post-operative setting—between March 2013 and July 2015. Patients included in this analysis had at least 6 months follow up or until their time of death if this was <6 months. SBRT was delivered with a particular focus on sparing organs at extreme risk (OAR_*Extreme*_), i.e., organs that approached their maximal radiation tolerance after the initial course of radiation and had a high potential to significantly impair a patient's QoL if toxicity were to occur in that site. Patients were simulated with standard immobilization of a thermoplastic head and neck mask. Simulation CT without IV contrast was obtained with a 2-mm slice thickness and MRI and/or PET images were fused at the discretion of the treating physician. In addition, delivered dose plans from the initial course of radiation were deformably registered to the new CT (Figure 1) using the deformable image algorithm from Velocity^TM^. The gross tumor volume (GTV) was defined by gross tumor seen on imaging studies and/or clinical exam. All gross disease i.e., primary and nodal recurrence, was treated. In the setting of postoperative radiation, the tumor bed (based on preoperative imaging, preoperative physical exam/endoscopy, operative findings, pathologic findings) plus region(s) of grossly involved lymphadenopathy was defined as the clinical target volume (CTV). No additional margin was added for the planning target volume (PTV) in order to limit toxicity. Doses of 40 Gy and 35 Gy were prescribed in the definitive or post-operative setting, respectively. Daily cone-beam CT and kV imaging was used in order to account for inter-fractional variations. The prioritized OAR_*Extreme*_ were dependent on the site of re-treatment: the cranial nerve foramen and skull base bony structures were prioritized in patients receiving radiation to skull base recurrences. In patients receiving radiation to the aerodigestive tract (AD), special attention was paid to the laryngeal cartilage and mandible. With lateral neck radiation, the brachial plexus and carotid arteries were prioritized. The distribution of doses to the OAR_*Extreme*_ were estimated using the deformably registered doses on the CT simulation scan. The total allowable doses to the OAR_*Extreme*_ were based on the EMAMI tables and RTOG head and neck protocols and calculated to its biologically equivalent dose using an α/β ratio of 3 (BED3) to estimate the risk of late toxicities to the normal tissues. See Table 1 for a list of prioritized constraints. Given the palliative intent of treatment, meeting OAR constraints took priority over PTV coverage in instances where both were not possible.

**Table 1:** Constraints.

**OAR**	**Constraint**
PTV	D95% = 98–100%
Larynx	Mean dose < 15 Gy
Mandible	Max dose < 20 Gy
Spinal cord/brainstem	Cumulative max dose < 54 Gy equivalent in 2 Gy/fx Or Max dose < 8 Gy (if records not available)
Cochlea	Mean dose < 15 Gy
Retina	Mean dose < 15 Gy
Lens	Max dose < 5 Gy
Carotid aa	Max dose < 32.5 Gy
Optic nerve/chiasm	Max dose < 25 Gy
Temporal tips	Mean dose < 5 Gy
Skin	D (10 cc) < 39.5 Gy
Thyroid lamina	Max dose < 30 Gy

Treatment was delivered twice a week for a total of five fractions via volumetric art therapy (VMAT) with cone-beam CT imaging before each treatment and in between each delivered arc. During image-guided treatment, the OAR_*Extreme*_, was used as the fusion surrogate. Concurrent systemic therapy was delivered at the discretion of the treating medical oncologist.

Our goal was to evaluate the efficacy and toxicity of treatment with OAR_*Extreme*_-sparing SBRT, as well as the effects of treatment on patient-reported QoL. QoL was assessed with several validated questionnaires: the M. D. Anderson Dysphagia Inventory (MDADI), the Symptom Inventory—Head and Neck Module (MDASI-HN), and the Xerostomia Questionnaire. Patients were asked to fill out all three questionnaires at their initial consult and subsequent follow up visits. The MDADI includes 20 questions that assess a patient's global, functional, emotional, and physical status. Scores range from 0 to 100, where 100 represents no impairment within the subscale. The MDASI-HN assesses the severity of a range of symptoms, including pain, fatigue, sadness, swallowing, speaking, nausea and vomiting, and insomnia, where each symptom can be rated from 0 to 10 (10 representing the most severe symptoms). The second section of the MDASI-HN assesses overall QoL by addressing symptom interference with daily function. Each question was also scored from 0 to 10, with 10 representing complete interference. The Xerostomia Questionnaire assesses difficulty talking, chewing, and swallowing due to dryness, as well as mouth/throat dryness during particular activities and the need for drinking liquids.

Patients were seen on treatment every week with acute toxicities scored based on the CTCAE 4.0 scale. Follow up after treatment was performed around 4–6 weeks, unless the patient had acute side effects that required earlier follow up. Regular follow up with the medical oncologist and/or head and neck surgeon was also recommended. CT or MR imaging was ordered 6 weeks after radiation completion and every 3 months afterwards for surveillance. PET/CT imaging was ordered if clinically indicated. Late toxicities were assessed at follow up appointments and scored based on the RTOG schema.

### Statistical Methods

Overall survival and time to progression, and time to progression or death (each measured from completion of SRS) were calculated using the Kaplan-Meier Method with statistical analyses performed in SPSS. *P*-values <0.05 were considered significant.

## Results

### Patient and Treatment Characteristics

A chart review of patients treated with SBRT in our department between the dates of November 2012 and July 2015 revealed 60 patients with a total of 69 sites treated (one patient with two sites treated concurrently and all eight additional sites were treated after a second failure). All patients had at least 6 months of follow up unless death occurred before 6 months. The median follow up time was 9.3 months.

Baseline patient demographics are summarized in Table 2. Thirty two (53%) patients underwent surgery for tumor recurrence. Thirty nine (57%) of patients received concurrent systemic therapy with SBRT, with a combination carboplatin and paclitaxel as the most frequently delivered regimen. Fifty one (74%) of the treated tumors were squamous cell carcinoma. Other histologies included adenocarcinoma, adenoid cystic carcinoma, basal cell carcinoma, thyroid carcinoma, and sarcoma. Patients with squamous cell carcinoma were treated with concurrent cetuximab at the discretion of the medical oncologist. Patients were divided into three categories based on the anatomical site of recurrences, for more specific reporting of treatment-related toxicities and QoL. These sites were the aerodigestive tract (43%), lateral neck (22%), and skull base (35%). If recurrence involved overlap of multiple groups, patients were included in the group in which the largest volume of disease was treated. Treatment of the nasopharynx was defined as skull base. Despite prioritizing OAR constraints over PTV coverage, the median D90 was 99.0% and the median V90 was 98.4%.

**Table 2:** Characteristics of patients treated with OAR_*Extreme*_-sparing SBRT.

**Patient characteristics**	**Number**
Total patients	60
Total sites treated	69
Patients with regional recurrence requiring re-SBRT	9
Gender	
Male	39 (70%)
Female	21 (30%)
Median age in years (range)	70 (48–88)
Median initial radiation dose in Gray	63.6
Median interval from prior irradiation in months	16.5
Surgery at recurrence	32 (53%)
Concurrent systemic therapy with SBRT	39 (57%)
Carboplatin/paclitaxel	20
Cetuximab	6
Carboplatin/paclitaxel/cetuximab	1
Cisplatin	3
Carboplatin/pemetrexed	2
Other/unknown	7
Histology	
Squamous cell carcinoma	51 (74%)
Adenocarcinoma	4 (6%)
Other	14 (20%)
SBRT site	
Aerodigestive tract (AD)	30 (43%)
Lateral neck (LAT)	15 (22%)
Skull base	24 (35%)
SBRT dosimetry	
Median treatment volume in cc	61.0
Median V90	98.4%
Median D90	99.0%
Median follow-up in months	9.3

### Tumor Control and Survival

**Figure 2:**
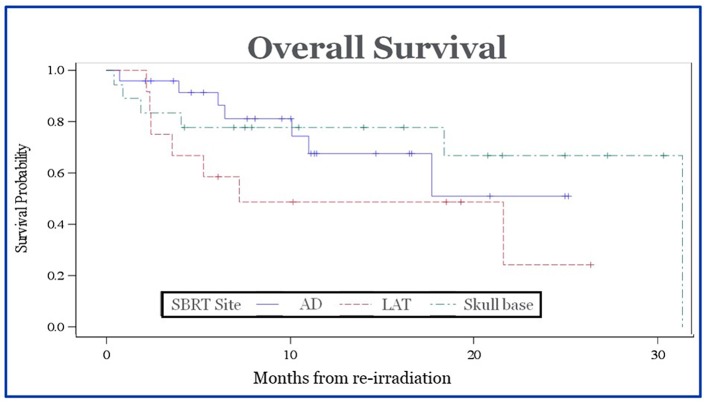
Overall survival of patients after completion of SBRT. There was no statistically significant difference in survival between groups based on site of recurrence.

Tumor control was assessed based on imaging and clinical findings. CT and/or MR imaging was routinely performed for follow up with PET/CT used for suspicious radiographic findings. The 1- and 2- year rates of local control were 79 and 79%, respectively. The median time to local failure was 43 months. The 1- and 2- year rates of regional control were 74 and 70%. The median time to regional failure was 36 months. The 1- and 2- year rates of distant control were 74 and 71%. The median time to distant failure was 36 months. The 1- and 2-year rates of overall survival were 59 and 45%. The median survival was 23 months. There was no statistically significant difference in median survival for patients who received chemotherapy at the time of recurrence vs. those who did not (12.93 mo vs. 11.33 mo, *p* = 0.82), or those who underwent surgery for their recurrent disease vs. no surgery (10.93 mo vs. 12.97 mo, *p* = 0.77). There was no difference in overall survival between groups based on anatomic location (*p* = 0.58) (Figure 2). There were no significant differences in local control or overall survival based on the site of irradiation.

### Second Recurrences

Eight patients had second recurrences of cancer within the initially irradiated field, with or without overlap onto the field receiving SBRT, and received re-irradiation with SBRT to a second site. The prescribed dose for the second course of re-irradiation was the same as primary re-irradiation, with 40 Gy prescribed in the definitive setting and 35 Gy in the post-operative; however, doses could be lowered if normal tissue tolerances could not be met. The median survival after re-treatment with SBRT was 6.57 months (range 1.40–21.57 months).

### Toxicity

Three patients (4%) experienced grade 3 late toxicity, including osteoradionecrosis (1 patient), chondroradionecrosis (1 patient), and osteomyelitis (1 patient). Two of the toxicities occurred in patients being treated to the aerodigestive tract, while one occurred in a skull base retreatment. There were no other variables significantly associated with toxicity rates. No grade 4 or 5 late radiation toxicities were observed in the study group.

### Quality of Life

**Figure 3:**
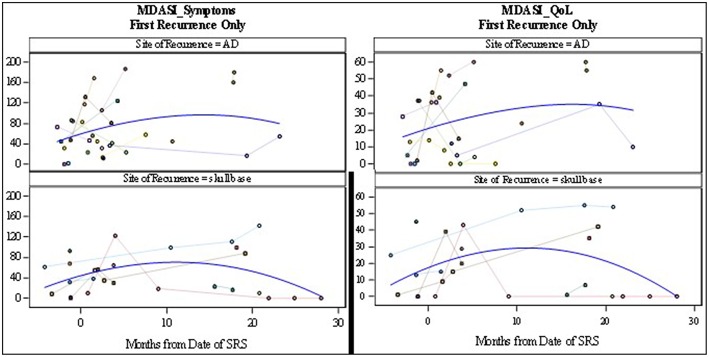
MD Anderson Symptom Inventory Scores: symptoms scores (**Left**) and Quality of Life scores (**Right**). Symptom scores range from 0 to 200 where 200 represents the most symptom interference with daily life. Quality of life scores range from 0 to 60 where 60 represents the most interference of symptoms on quality of life metrics.

**Figure 4:**
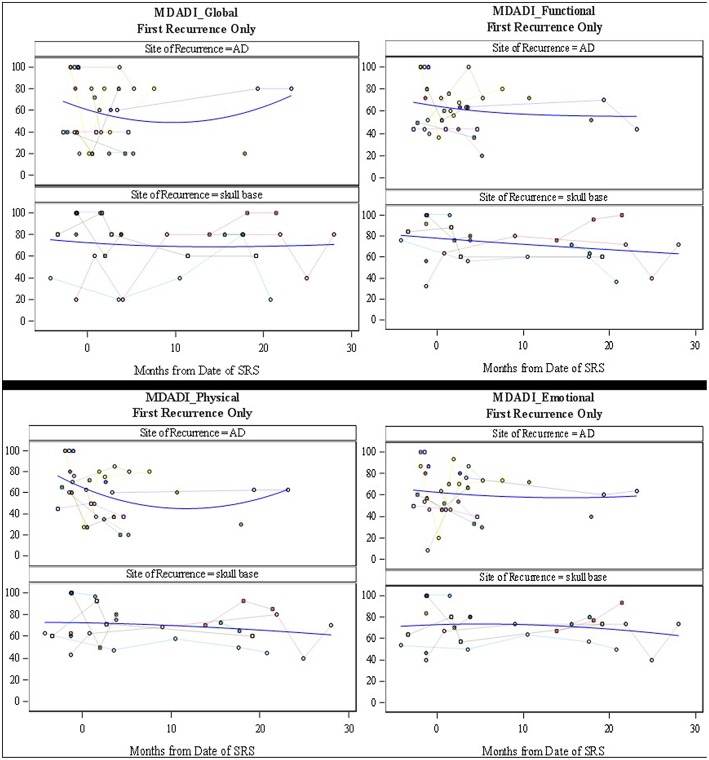
MD Anderson Dysphagia Inventory Subscale Scores (Global, Functional, Physical, and Emotional). Scores range from 0 to 100 where 100 represents the least interference with daily life. Individual patient scores (faint colors) and smoothed fitted curve (blue) are shown.

The MDADI responses demonstrated that patients with re-irradiation to the skull base, maintained stable dysphagia-associated scores from pre-treatment to long-term follow up. Patients with retreatment to the aerodigestive tract demonstrated a slight decrease in global scores, correlating with the increase in symptoms seen on the MDASI, before returning near baseline. The MDASI-HN showed an increase in patient-reported symptoms in the acute time period after treatment in the skull base group, with a longer period to symptom development in patients treated to the aerodigestive tract. The QoL scores assessed with this metric followed closely with symptoms cores with a small decrease in QoL before returning closer to baseline (Figures 3, 4). Patients in all three groups demonstrated an increase in xerostomia scores after treatment; however, there was a trend to an improvement in xerostomia patients in patients in the skull base group in long term follow up. There were not enough patients with re-irradiation to the lateral neck who completed pre- and post-treatment questionnaires to analyze this site-specific QoL data. There were no other captured variables associated with QoL metrics, such as use of chemotherapy, volume irradiated, or use of surgery before SBRT.

## Discussion

Locoregional control of head and neck cancer continues to remain a primary challenge in head and neck cancer with half of patients dying of localized disease (8, 9). Resection of localized recurrence has demonstrated long-term local control rates of 25–45%; however, over half of these patients will recur locally (10–13). Patients at a high risk of recurrence, such as with residual disease or extracapsular extension of nodal disease, may be recommended to undergo postoperative re-irradiation. In a phase III study patients with recurrent disease in a previously-irradiated field underwent surgery followed by either conventionally fractionated chemoradiation or observation. Disease free survival in the adjuvant radiation group was improved; however, significant high grade toxicities were observed (28% patients with grade 3 and 39% with late grade 4 toxicities) with no improvement in overall survival (14). This study serves as the foundation for recommending adjuvant chemoradiation but demonstrates the high rate of morbidity associated with treatment.

For non-surgical candidates, definitive chemoradiation can be offered with the intent to obtain long term tumor control. Re-irradiation with conventional fractionation to a dose of 60 Gy in 40 BID fractions, as per RTOG 9610, resulted in nearly a 20% rate of late grade 3 toxicities and 3% late grade 4 toxicities. The 1- and 2- year overall survival rates of this group were 42 and 17% with a median survival of 8.5 months (2). Compared to 3D techniques, IMRT has improved the rate of local control in the re-irradiation setting (52% vs. 20% at 2 years); however, even with IMRT, the doses that can be delivered are significantly limited by constraints set by the prior course of radiation. With advances in imaging and image guided radiation therapy, larger doses per fraction and smaller margins can be used, and SBRT has been shown in retrospective studies to improve local control. Phase I and II trials have also demonstrated the safety of using SBRT up to a dose of 44 Gy in 5 fractions in the re-irradiation setting, as well as decreased toxicity in comparison to historical data from conventional radiotherapy (15).

Previously published outcomes of SBRT for retreatment have demonstrated local control rates of 30–80% at 1–2 years with overall survival rates of 20–60% at the same time interval (5, 16). Vargo et al. published data on the use of adjuvant re-irradiation with SBRT for patients with positive margins and/or extranodal extension and found a 1 year locoregional control rate of 51% and survival rate of 64% (17). The rates of late severe toxicities were lower than with conventional re-irradiation, with 8% experiencing grade 3 or higher toxicities. Our data shows comparable rates of local control as compared to previous studies of SBRT for recurrences; however, patients in our group were treated to substantially larger tumor volumes, which have been correlated with decreased tumor control (18).

Recurrences after SBRT for re-irradiation are not uncommon, with reported rates up to 59% in the literature (5). In the series presented by Vargo et al., 23% of the patients who were re-irradiated with SBRT and concurrent cetuximab developed a nodal recurrence, for which they were again re-irradiated with SBRT (17). In our series, 15% of patients received repeat SBRT for locoregional recurrences, with a median survival of over 6 months after their second course of retreatment. As patient survival increases and local treatment improves, regional failures begin to play a larger role in long-term tumor control and symptoms. Repeat SBRT for re-irradiation has not been widely performed and longer-term data is needed to address the safety and toxicity associated with multiple courses of hypofractionated radiation; however, if patients are to be candidates for repeat re-irradiation, then extra caution should be applied to the normal tissues, as the risks for radiation-induced injury increase.

The incidence of late toxicities from re-irradiation with SBRT are substantially lower than those seen with older techniques; however, there are still significant and life-threatening toxicities associated with treatment. A recently published 10-year update of toxicities from SBRT re-irradiation showed 18.9% of patients experiencing late grade ≥3 toxicities, with significantly higher rates of severe toxicity in patients retreated to the larynx/hypopharynx and when doses ≥44 Gy were used (19). One of the most morbid toxicities associated with re-irradiation is carotid blow-out syndrome (CBOS), which was seen in 3–17% of patients retreated with SBRT and usually presents within 10 months of retreatment (5). Yazici et al. also published their institutional experience with carotid blow out syndrome and demonstrated that treatment every other day and doses <34 Gy reduce the rate of CBOS (20). At our institution, the total allowable maximum dose to the carotid was 32.5 Gy, with treatments delivered twice a week, and no patients within our cohort developed a carotid blow out. We would propose that this constraint should be strictly followed even if this compromises PTV coverage.

The toxicities from radiation have also been shown to play a significant role in decreasing the QoL in patients undergoing palliative treatment. Irradiation to the head and neck with conventional techniques has been shown to cause significant mucositis and dysphagia, causing pain, and decreased oral intake (2, 7). In addition, conventional fractionation is associated with facial and neck edema. This was not observed in patients receiving SBRT in our cohort. This was likely due to our attempt to limit the amount of sub-parenchymal normal tissue in our field, in addition to the lack of margin added to GTV and the use of only twice-weekly radiation. Even with the use of SBRT in delivery of re-irradiation, patient reported QoL decreased in up to 50% of patients (15). However, there seems to have been an improvement in treatment delivery as recent publications show unchanged QoL scores after treatment (17). In our study, patient-reported QoL data was collected at the time of consultation and in follow up. While the data appears to demonstrate an increase in symptoms in the first month after treatment, the MDADI-assessed QoL assessed appears to remain stable in both short- and long-term follow up. It is important to note that even as the spline shows stability of scores (Figures 3, 4), individual patient responses were quite varied with some patients demonstrating dramatic improvements while others experiences a long-term decrease in QoL.

Our study has several important limitations. First, it is a retrospective study on a non-randomized cohort of patients from a single institution, treated by a single physician. Second, despite only including patients who had at least 6 months follow up, the median follow up time was <1 year, which is not entirely enough time to rule out the development of any further grade 3 and higher toxicities. Several papers evaluating the use of SBRT in the setting of re-irradiation have described a higher rate of severe toxicities after longer term follow up and one publication describes a continuous increase in the rate of late toxicities more than 5 years after treatment (19, 21, 22). The low rate of late toxicities seen in this population are promising, but significantly longer follow up of long-term survivors is important to evaluate for tissue damage associated with re-irradiation. Third, not all patients completed the MDADI, MDASI, and/or XQ prior to and after treatment. Patients who completed the surveys may represent a selection bias. This potential bias, as well as the inherent problems of reliability in intra-rater perception, is important to consider in evaluating the QoL data.

## Conclusion

Based on the results seen in this retrospective study, OAR_*Extreme*_-sparing SBRT is able to achieve comparable local control and overall survival rates as previously published data for re-irradiation of head and neck cancer. Despite prioritizing meeting the constraints of the OAR_*Extreme*_ over PTV coverage, high quality plans with excellent coverage of the PTV were able to be created. The potential for lower toxicities with maintained tumor control and QoL using OAR_*Extreme*_-sparing SBRT provides a promising salvage treatment for patients with in-field recurrences of head and neck cancer and preserves a treatment option of repeat re-irradiation if patients develop a second in-field recurrence.

## Data Availability

The datasets generated for this study are available on request to the corresponding author.

## Author Contributions

EG and IZ both provided contributions including chart mining, statistical analyses, and writing. ZR provided statistical analysis and contributed to writing. MM, GG, AS, and AR all provided physics contributions, include dosimetric analyses and figure production. ST and MG treated the majority of patients and provided senior authorship duties such as oversight of the project as a whole.

### Conflict of Interest Statement

The authors declare that the research was conducted in the absence of any commercial or financial relationships that could be construed as a potential conflict of interest.
